# Mexican Propolis: A Source of Antioxidants and Anti-Inflammatory Compounds, and Isolation of a Novel Chalcone and ε-Caprolactone Derivative

**DOI:** 10.3390/molecules23020334

**Published:** 2018-02-06

**Authors:** Silvia Laura Guzmán-Gutiérrez, Antonio Nieto-Camacho, Jorge Ivan Castillo-Arellano, Elizabeth Huerta-Salazar, Griselda Hernández-Pasteur, Mayra Silva-Miranda, Omar Argüello-Nájera, Omar Sepúlveda-Robles, Clara Inés Espitia, Ricardo Reyes-Chilpa

**Affiliations:** 1 Departamento de Inmunología, Catedrática CONACyT-Instituto de Investigaciones Biomédicas, Universidad Nacional Autónoma de México, Ciudad Universitaria, Coyoacán 04510, Ciudad de México, Mexico; laura.guzman@iibiomedicas.unam.mx (S.L.G.-G.); mayra_1203@yahoo.com.mx (M.S.-M.); 2 Departamento de Productos Naturales, Instituto de Química, Universidad Nacional Autónoma de México, Ciudad Universitaria, Coyoacán 04510, Ciudad de México, Mexico; camanico2015@yahoo.com (A.N.-C.); lizquim2@unam.mx (E.H.-S.); yolotzin_gris@hotmail.com (G.H.-P.); 3 Departamento de Farmacobiología, CINVESTAV-SUR, Instituto Politécnico Nacional, Calzada de los Tenorios 235, Col. Granjas Coapa C.P. 14330, Ciudad de México, Mexico; jorge.ivan.df@gmail.com; 4 El Colegio de la Frontera Sur, Ecosur-San Cristóbal, Carretera Panamericana y Periférico Sur s/n Barrio María Auxiliadora, San Cristóbal de Las Casas C.P. 29290, Chiapas, Mexico; arguello@ecosur.mx; 5 Catedrático CONACyT—Unidad de Investigación Médica en Epidemiología Clínica, Hospital de Pediatría, Centro Médico Nacional Siglo XXI, Instituto Mexicano del Seguro Social (IMSS). Av. Cuauhtémoc No. 330. Colonia Doctores, Delegación Cuauhtémoc C.P. 06720, Ciudad de México, Mexico; sero__82@hotmail.com; 6 Departamento de Inmunología, Instituto de Investigaciones Biomédicas, Universidad Nacional Autónoma de México, Ciudad Universitaria, Coyoacán 04510, Ciudad de México, Mexico

**Keywords:** Mexican propolis, red propolis, epoxypinocembrin chalcone, ε-lactone, antioxidant activity, anti-inflammatory activity

## Abstract

The propolis produced by bees are used in alternative medicine for treating inflammation, and infections, presumably due to its antioxidant properties. In this context, five propolis from México were investigated to determine their inhibitory lipid peroxidation properties. The ethyl acetate extract from a red propolis from Chiapas State (4-EAEP) was the most potent (IC_50_ = 1.42 ± 0.07 μg/mL) in the TBARS assay, and selected for further studies. This extract afforded two new compounds, epoxypinocembrin chalcone (**6**), and an ε-caprolactone derivative (**10**), as well as pinostrobin (**1**), izalpinin (**2**), cinnamic acid (**3**), pinocembrin (**4**), kaempherol (**5**), 3,3-dimethylallyl caffeate in mixture with isopent-3-enyl caffeate (**7a** + **7b**), 3,4-dimethoxycinnamic acid (**8**), rhamnetin (**9**) and caffeic acid (**11**). The HPLC profile, anti-mycobacterial, and antioxidant properties of this extract was also determined. Most of the isolated compounds were also tested by inhibition of reactive oxygen species (ROS) in challenged mouse bone marrow-derived mast cells (BMMCs), and DPPH. Their anti-inflammatory activity was evaluated by TPA, and MPO (myeloperoxidase) activity by ear edema test in mice. The most potent compounds were **7a** + **7b** in the TBARS assay (IC_50_ = 0.49 ± 0.06 μM), and **2** which restored the ROS baseline (3.5 μM). Our results indicate that 4-EAEP has anti-oxidant, and anti-inflammatory properties due to its active compounds, suggesting it has anti-allergy and anti-asthma potential.

## 1. Introduction

Propolis is a resinous product made by bees from plant exudates or resins that bees mix with wax and their salivary secretions. The bees use the propolis as a building material to reduce the entry at their hives, to repair cracks, and strengthen the walls, mainly at the edges. They also use it to varnish the interior of their hive, in order to avoid proliferation of pathogenic microorganisms and, to embalm the corpses of intruder animals that are not possible to remove due to their large sizes, avoiding in this way their putrefaction [1].

Scientific studies are shown that propolis possess pharmacological activities such as anti-oxidant, anti-inflammatory, anti-viral, anti-fungal, immunomodulator, anti-cancer, local anesthetic, hepatoprotector, radioprotector, chemopreventive, and even as a neuroprotector [1,2,3]. The antimicrobial activity of propolis has been known since ancient times; therefore, it has been integrated into the medicinal products heritage. However, the pharmacological effects of propolis are strongly marked by their composition and depends on the place where these were collected, the type of vegetation, season and species of bee [4,5,6]. Thus, the propolis are very variable and complex, differing in their chemical composition and properties; for instance, it is possible to find propolis of different colors, flavors, and textures. 

The great diversity of propolis demands that during their study, to take in account its geographical origin and even season of collection [7,8,9,10]. European propolis contains flavonoid aglycones, phenolic acids and their esters [11]. In Brazilian propolis there was found prenylated derivatives of *p*-coumaric acid and acetophenone [12], diterpenes, lignans and flavonoids [13]. The main components of Cuban propolis are polyisoprenylated benzophenones [14]. From Chilean propolis 17 compounds were isolated that belong to the phenylpropane, benzaldehyde, dehydrobenzofuran, or benzopyran classes [15]. Among the isolated compounds from propolis, pinostrobin (**1**) is the most studied. It has been demonstrated that this flavanone has anti-inflammatory and neuroprotective effects and the ability to reduce ROS, modulate mitochondrial function, and regulate apoptosis, also can protect the brain against damage from ischemic stroke [16]. The flavonoids chrysin and kaempferol (**5**) have been identified as responsible for the anti-allergic effect of Chinese propolis. Other common components of propolis, such as caffeic acid and caffeic acid phenethyl ester, induce apoptosis in human tumor cells in vitro [17]. 

In Mesoamerica the culture of *Melipona beecheii* (bee without sting) and consumption of honey was a common practice in pre-Columbian times. After the conquest of México in 1521, the beekeeping with the European bee (*Apis mellifera*) was also developed in several regions. Nowadays, México is one of the main honey bee producers in the world; the States of Yucatán, Campeche, Jalisco and Chiapas are the major contributors. In the case of Mexican propolis, different type of compounds have been isolated, such as, phenylallylflavanones [18,19], 1,3-diarylpropane, 1,3-diarylpropene derivatives, flavanones, isoflavans, and pterocarpans [20]. 

In this work, we assessed the inhibition of lipid peroxidation by TBARS assay of five propolis samples of *Apis mellifera* from the States of Chiapas, and Yucatán, México. The sample 4 from Chiapas State was a red propolis, and its ethyl acetate extract (4-EAEP) was the most potent in the TBARS assay and therefore selected for further chemical and pharmacological studies. From this extract two new and ten known compounds were isolated. We also assessed the anti-inflammatory, anti-oxidant and anti-mycobacterial activities of this extract and its majoritarian compounds.

## 2. Results

### 2.1. Screening of Antioxidant Activity

Five Mexican propolis were obtained from the States of Chiapas, and Yucatán, and examined for their antioxidant activity in the TBARS assay (Table 1). Only the samples 2, 3, and 4 (4-EAEP) showed activity at 10 µg/mL, and their IC_50_ were calculated. The most potent extract 4-EAEP with IC_50_ = 1.42 ± 0.07 μg/mL was selected for further chemical and pharmacological studies. 

### 2.2. Chemical Analysis of the Most Potent Extract

**Figure 1:**
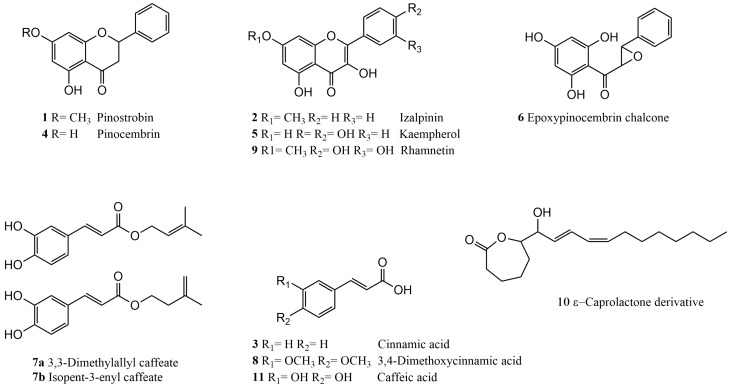
Chemical structures of compounds isolated from propolis extract.

The 4-EAEP was subjected to CC (40 g) and afforded 12 compounds (Figure 1). Two new compounds, epoxypinocembrin chalcone (**6**), and a ε-caprolactone derivative (**10**) have not been previously reported, and their identification is presented next.

#### 2.2.1. Structure Elucidation of Epoxypinocembrin Chalcone (**6**)

The ^1^H-NMR revealed five aromatic protons as two signals at δ 7.53 (2H, dd *J* = 2 Hz, 2.1 Hz) and δ 7.41 (3H, m), indicating a flavonoid monosubstituted aromatic ring. In addition, two doublets at δ 5.94 (1H, d, *J* = 2.1 Hz) and δ 5.90 (1H, d, *J* = 2.1 Hz) were assigned to two *meta*-coupled protons of the aromatic A ring, therefore this must be tetrasubstituted. A pair of doublets at δ 5.07 (1H, d, *J* = 11.55 Hz) and δ 4.54 (1H, d, *J* = 11.58 Hz) indicated two methines on a carbon attached to oxygen, and the coupling constant indicated these pair of hydrogens must be in the *ci*s position. 

**Figure 2:**
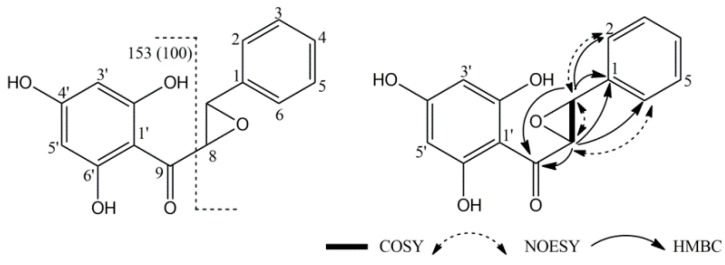
Structure of epoxypinocembrin chalcone **6** and selected EI-MS fragments, and NMR correlations.

The combined analysis of its ^13^C and HSQC spectrums revealed the presence of 15 carbons assigned to nine methines (seven aromatic and two bonded to oxygen) and six quaternary carbons (one carbonyl and five aromatic). With this information we initially proposed a one tetrasubstituted benzene ring attached to a monosubstituted benzene ring through a dihydroxypropanone chain. However, the high resolution mass spectral data by ESI-MS (positive mode) indicated a [M + H]^+^ ion at *m*/*z* 273.07 suggesting the molecular formula C_15_H_13_O_5_, therefore our proposal had an extra hydroxyl group. In addition, IE-MS showed the base peak with *m*/*z* 153, that corresponds to the molecular ion trihydroxybenzonium minus 119 (Figure 2), in agreement with a phenyloxiranyl group [21,22]. This suggested that the compound had an epoxychalcone structure (Figure 2). The J^2^, and J^3^ connections C-H were determined from the HMBC spectrum, the carbonyl carbon C-9 showed correlations with H-7 and H-8; at the same time C-1 show cross peaks with H-7 and H-8; and C-7 with H-2 and C-6, indicated a phenyloxyranyl group attached to a substituted phenyl ketone. NOEs enhancements can be observed via the 2D NOE technique (NOESY). NOE between H-7/H-8/H-2/H-6. The final proposal is depicted (Figure 2) and the complete assignments of ^1^H- and ^13^C-NMR are shown in Table 2. 

#### 2.2.2. Structure Elucidation of the ε-Caprolactone Derivative **10**

The DART mass spectrum indicated the molecular formula C_18_H_30_O_3_, and four degrees of unsaturation. The EI mass spectrum showed the fragments pattern of allylic chain present in the molecule. The ^13^C-NMR (175 Hz) revealed 18 signals and in agreement with HSQC and HMBC experiments there were assigned to one carbonyl, one methyl, 10 methylene_,_ and six methine groups. The carbonyl group (δ 179.9, C-1) was one of the four unsaturations of the molecule, while the IR spectrum suggested the presence of a lactone ring (1707 cm^−1^). In addition in the ^13^C spectrum four olefin methines signals were noticed at δ 133.53, 128.37, 129.43 and 133.08 (C-8, C-9, C-10 and C-11, respectively); the HSQC spectrum showed the corresponding ^1^H spectrum signals at δ 5.73, 6.56, 6.00 and 5.41 (H-8, H-9, H-10 and H-11, respectively). The coupling constants and the HMBC experiment revealed the diene system (H-11/C-9 and H-8/C10). The values of the coupling constant between H-8 (δ 5.75, *J* = 15.7 Hz) and H-9 (δ 6.56, *J* = 15, 11.1) indicated the presence of *trans*-olefinic protons, while that between H-10 (δ 6.0, *J* = 11.1, 11.1) and H-11 (δ 5.41, *J* = 11, 7.7) showed a *cis*-double bond. The TOCSY spectrum reveled that H-8 was coupled to an allylic methine proton at δ 3.96 (H-7) indicating the presence of an allylic alcohol (IR, 3266.55 cm^−1^), which in turn is also coupled to another methine in δ 3.48 (H-6) attached to an oxygen, and consecutively with two additional methylenes: H_2_-5 (δ 1.34) and H_2_-4 (δ 1.53), while on the other side of the molecule, H-11 has coupling with a methylene at δ 2.19 (H_2_-12) and a methylene at δ 14.4 (H_3_-18) [23,24].

**Figure 3:**
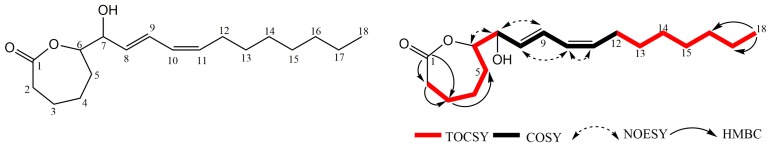
Structure and selected NMR correlations of ε-caprolactone derivative.

The IR spectrum showed an absorption band at 1707 cm^−1^ which was assigned to the carbonyl of a seven member lactone on basis of the following evidences. In the HMBC spectrum correlations between the carbonyl carbon C-1 with the protons of two methylene groups H_2_-2 (δ 2.22) and H_2_-3 (δ 1.6) were observed the at the same time H_2_-3 had a correlation with C-5 (δ 30.5). The signals observed in the TOCSY experiment also supported this idea, in so much as H-2 had correlation with H-3 (δ 1.59), H-4 (δ 1.53) and H-5 (δ 1.34). On the other hand, the NOE correlations between H-10/H-8/H-11 and H-7/H-9/H-6 were observed. Thus, the structure of **10** was determined as shows in the Figure 3. The complete assignments of ^1^H- and ^13^C-NMR spectra are given in Table 3. 

#### 2.2.3. HPLC Profile of 4-EAEP

**Figure 4:**
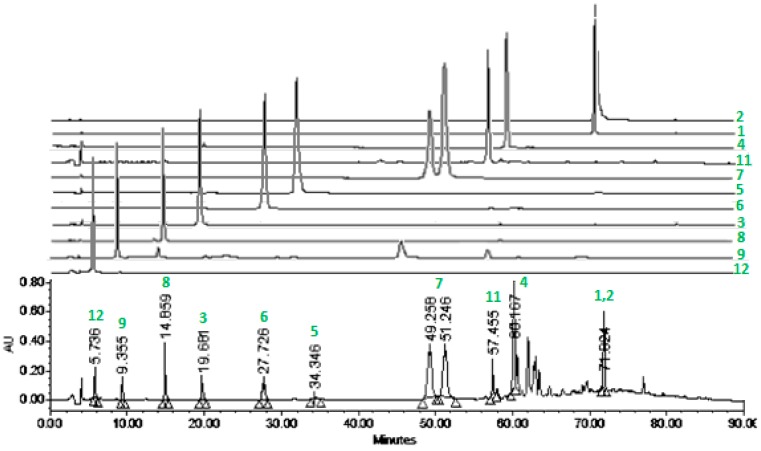
Chromatogram of propolis extract (5 mg/mL, 20 µL injection) at 236 nm. (**1**) (λ_max_ 289 nm), (**2**) (λ_max_ 266.6 nm), (**3**) (λ_max_ 277.2 nm), (**4**) (λ_max_ 290.2 nm), (**5**) (λ_max_ 266.6 nm), (**6**) (λ_max_ 291.4 nm), (**7**) (λ_max_ 327 nm), (**8**) (λ_max_ 322.2 nm), (**9**) (λ_max_ 323.4 nm), (**10**) (λ_max_ 236 nm), (**11**) (λ_max_ 324.6 nm).

The HPLC chromatogram of 4-EAEP (Figure 4) allowed us to observe the complete composition of propolis extract, and each of the isolated compounds, indicating the method is helpful for monitoring their presence in other samples. The chromatogram confirmed that the most abundant compounds are the flavonone pinocembrin (**4**) followed by the flavonol pinostrobin (**1**), the mixture of prenylated phenylproponoids (**7a** + **7b**) and the flavonol izalpinin (**2**), while the new compounds **6** and **10** were the scarcest.

### 2.3. Biological Activity

#### 2.3.1. TBARS

**Figure 5:**
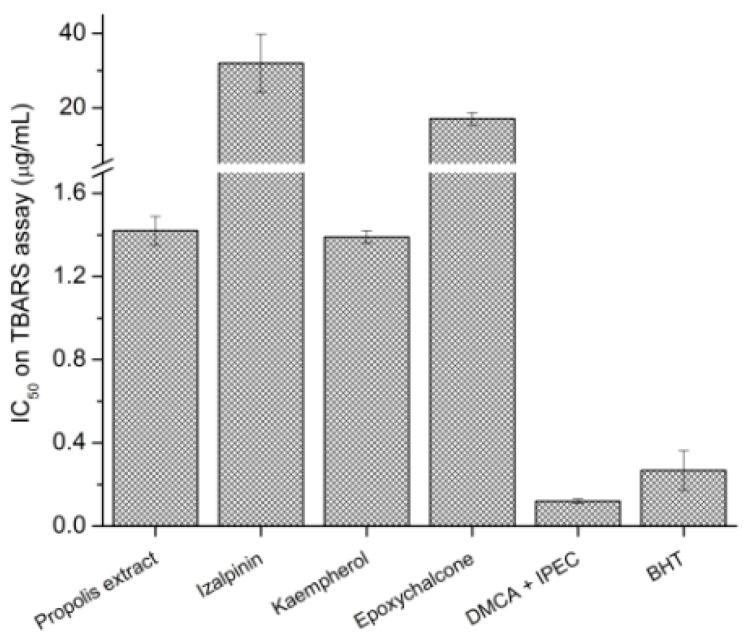
Comparison of IC_50_ levels of tested compounds isolated of propolis extract in TBARS assay. Data represent the mean ± SEM (*n* = 3).

In the TBARS assay 4-EAEP showed antioxidant activity in concentration-dependent manner with an IC_50_ = 1.42 ± 0.07 μg/mL, and was the most potent effect exhibited by this extract. The most active compounds were **7a** + **7b** IC_50_ = 0.49 ± 0.06 μM, followed by **5** IC_50_ = 4.87 ± 0.11 μM, **6** IC_50_ = 59.96 ± 6.4 μM, **2** IC_50_ = 112.78 ± 27.37 μM. In the Figure 5 the comparison of IC_50_ (µg/mL) of the different tested compounds with the propolis extract is shown. The positive control was BHT (IC_50_ = 1.22 ± 0.44 µM). The compounds **1**, **4** and **8** were not active.

#### 2.3.2. Determination of ROS in the Antigen-Induced Mast Cell Degranulation

**Figure 6:**
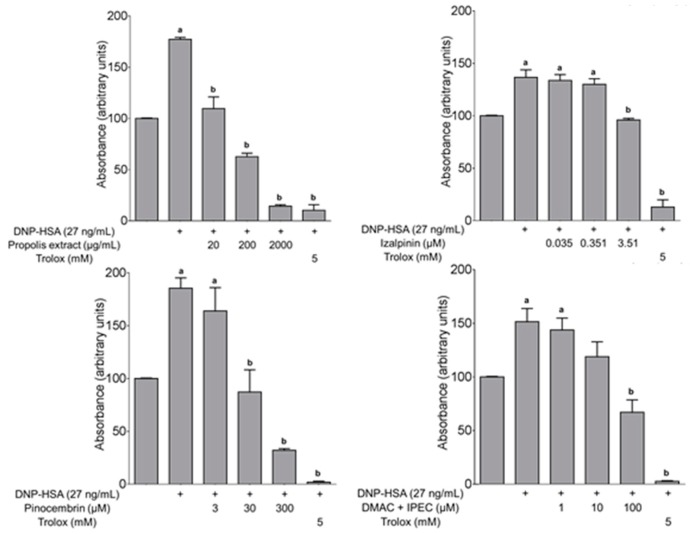
The inhibition of ROS production in stimulated mast cells. DMAC + IPEC mixture of 3,3-dimethylallyl caffeate and isopent-3-enyl caffeate (**7a** + **7b**). Data represent the mean ± SEM (*n* = 3). ANOVA followed by Tukey’s test, * *p* < 0.05. (**a**) Significant difference compared to the control group and (**b**) to the stimulated group. (+) Groups with DNP-HAS antigen.

The 4-EAEP at 20 μg/mL was able to restore ROS baseline associated to activation FcεRI by antigen, corroborating the presence of one or more compounds with antioxidant activity. The ROS inhibition of some purified compounds was tested (Figure 6). The compound **2** was the most potent, since 3.5 μM restore completely the ROS baseline, then followed by **4** (30 µM), **7a** + **7b** (100 µM), and finally the **8** (300 µM), the **1** was not active. 

#### 2.3.3. DPPH Scavenging Capacity

In the case of DPPH assay, the 4-EAEP had an IC_50_ = 16.55 ± 0.87 µg/mL. The compounds **7a** + **7b**, showed free radical scavenging activity, with an IC_50_ = 13.63 ± 0.2 µM, and **5** with an IC_50_ = 26.02 ± 0.08 µM. The positive controls were BTH (IC_50_ = 16.51 ± 1.27 µg/mL) and α-tocopherol (41.15 ± 0.14 µM). The **1**, **2**, **3**, **4**, **6** and **8** not had activity. 

#### 2.3.4. Anti-Inflammatory Effect and Inhibition of Myeloperoxidase

**Figure 7:**
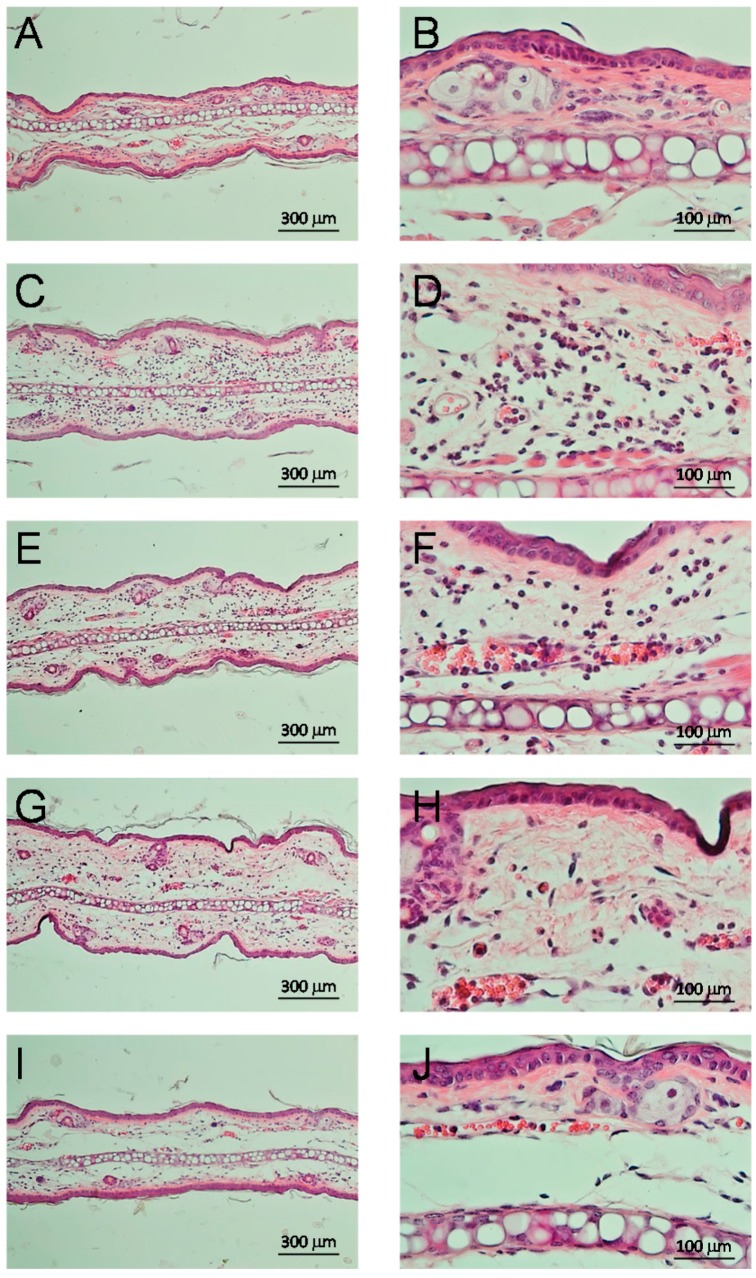
Representative histological sections from mice ears stained with hematoxylin-eosin (10×, left column and 40×, right column), after 4 hours of TPA application and 4-EAEP at different doses. (**A**,**B**) Basal; (**C**,**D**) TPA; 4-EAEP (**E**,**F**) 0.56 mg/ear; (**G**,**H**) 1 mg/ear and (**I**,**J**) 1.71 mg/ear.

The 4-EA EP showed in the TPA model an IC_50_ = 1.21 mg/ear, while for the positive control, indomethacin showed an IC_50_ = 0.84 mg/ear, indicating that the 4-EAEP had activity. Only the isolated pinocembrin (**4)** had activity, with an IC_50_ = 2.53 µmol/ear (0.64 mg/ear), while celecoxib had 0.91 µmol/ear. The histological sections from mice ears were shown in Figure 7, where we can see the 4-EAEP diminution of cells migration observed in the inflammation process. The 4-EAEP at 1 mg/ear produced a diminish of levels of myeloperoxidase at level of 95.46 ± 0.002%, as comparison, indomethacin at 0.358 mg/ear achieved inhibition of 91.08 ± 0.006%. Pinocembrin at 0.81 mg/ear produced 91.09 ± 3.66% of inhibition [25,26]. **1**, **2**, **3**, **5**, **6**, **7**, **8** and **9** were not active.

#### 2.3.5. Anti-Mycobacterial Activity

The anti-mycobacterial activity of 4-EAEP was evaluated and had a MIC = 250 µg/mL and the toxicity in the VERO cells was IC_50_ = 179 µg/mL. The isolated cinnamic acid (**3**) also presented a moderated activity with a MIC = 250 µg/mL.

## 3. Discussion

### 3.1. Screening

The extracts of five propolis samples from the Southeast States of México, Chiapas and Yucatán were analyzed for their antioxidant activity in the TBARS assay. Only the samples 2, 3, and 4, collected at the higher altitudes showed relevant activity. The most potent (IC_50_ = 1.42 ± 0.07 μg/mL) was 4-EAEP (Table 1) from a red propolis collected at San Cristobal de las Casas (2200 m), while those collected nearby to sea level were almost inactive. San Cristobal de las Casas predominant natural vegetation is pine-oak forests, and wetlands, agriculture is devoted to maize, but horticulture, floriculture, and cultivation of temperate fruit trees is also practiced. Pesticides are used in the case of floriculture [27]. 

### 3.2. Chemical Analysis and HPLC Profile of 4-EAEP

The extract 4-EAEP afforded 12 compounds, six flavonoids, five phenylpropanoids, and one fatty acid lactone (Figure 1). Two of the compounds are new: epoxypinocembrin chalcone (**6**), and an ε-caprolactone derivative **10**. The compound **6** could arise from the known pinocembrin chalcone, which has been previously isolated from propolis of Poland [28], Canada [29], among others. Regarding to compound **10**, it could arise from cyclization of polyunsaturated octadecanoic acid, it is well known that fatty acids are constituents of propolis of different regions [30]. 

The majoritarian compounds of 4-EAEP were the flavonones pinocembin (**4**), pinostrobin (**1**), the mixture of prenylated phenylproponoids (**7a** + **7b**), and the flavonol izalpinin (**2**) (Figure 4). The overall chemical composition of this extract resembled those from European propolis [11]. Previously it has been studied a Mexican red propolis from Champotón located at the nearby State of Campeche [20] isolating in addition to flavanones, isoflavans and pterocarpans characteristic of Papilionoideae plant species. Champotón is located at sea level, tropical vegetation may be the main reason for the differences in composition with our red propolis. Our results support the idea that geographic origin, altitude, natural vegetation, and predominant cultivated and ruderal plant species, determine the chemical composition of propolis and their pharmacological properties. 

### 3.3. Biological Activity

The 4-EAEP, and their compounds isolated in good yield, were tested for antioxidant activity, in the tree different tests (TBARS, DPPH and ROS), as well as for anti-inflammatory (TPA) and anti-mycobacterial properties. In general, the best results were obtained for antioxidant activity, specially inhibiting lipid peroxidation as evaluated by TBARS production, and inhibition ROS production in mouse mast cells, followed by scavenging activity of free radical DPPH. The extract and several compounds also showed anti-inflammatory activity. The 4-EAEP showed low anti-mycobacterial activity, and toxicity in the VERO was also low.

The most potent effect exhibited by 4-EAEP was inhibition of lipid peroxidation in a concentration-dependent manner. This assay measures through MDA the total lipid hydroperoxides content, formed in a later stage of lipid oxidation. The high activity of 4-EAEP in this model should be due to the activity of the majority of the isolated compounds. The mixture of isomers **7a** + **7b** was 2.4 times more potent that positive control BHT, followed by **5**, **6** and **2**. The lipid peroxidation can lead to cell membranes to lose their integrity, and therefore their functions Moreover, the aldehydes formed by lipid oxidation can react with proteins and nucleic acids, which determines cytotoxic, genotoxic and mutagenic effects, as well as a pathogenic role in several diseases [31].

The 4-EAEP was able to restore ROS baseline associated to activation FcεRI by antigen, corroborating the presence of one or more compounds with antioxidant activity. The compound **2** was the most potent, followed by **4**, **7a** + **7b**, and finally **8**. These results show the capacity of the isolated compounds to restore the equilibrium of ROS production at cell level; on the other hand, this is the first time that these compounds are tested in this model. In the case of mixture **7a** + **7b**, although these compounds have been indicated as topic allergens, this experiment shows that at a certain concentration they can help to reduce the oxidative stress in stimulate mast cells and the molecular mechanism is independent and different to that previously described [32,33]. The increase in ROS production by the molecular protein complex of NADPH oxidase in mast cells, after cross-linking of FcεRI with IgE/Ag, is associated with the importance of ROS for the activation of receptor-operated calcium entry (ROCE), which favors the entry of calcium through activated membranal calcium channel into the cell, resulting in the mobilization and release of the pro-inflammatory content of the preformed granules in mast cells [34]. This model simulates what happens in an acute inflammatory process observed in chronic degenerative diseases, such as allergies and asthma, where mast cells play an important role. Kaempferol (**5**), a component of 4-EAEP has been identified as an anti-allergic component on mast cells of the ethanol extract of a Chinese propolis [35]. Additionally, it has been investigated the effects of an aqueous extract of propolis (13%), administered as an adjuvant to therapy to patients with mild to moderate asthma [36]. At the end of the treatment, patients receiving propolis showed a marked reduction in the incidence and severity of nocturnal attacks and improvement of ventilator functions that was associated with decreases of prostaglandins, leukotrienes, pro-inflammatory cytokines (TNF-α, IL-6, IL-8) and increased IL-10.

In the case of DPPH assay, the EAEP had a similar activity to positive control BTH. Only the mixture of **7a** + **7b** showed free radical scavenging activity, and was three times more potent that α-tocopherol. Regarding to anti-inflammatory activity, pinocembrin (**4**) was active, but the extract showed a mild effect, probably because it is mixed with other substances. This is the first report of the anti-inflammatory effect of pinocembrin in TPA model in mice and its respective IC_50_. The histological sections from mice ears treated with 4-EAEP showed diminution of cell migration in the inflammation process. Related to this result, the enzyme myeloperoxidase is produced by neutrophils, and increases its activity when an inflammatory process is triggered by TPA administration. Pinocembrin (**4**) and 4-EAEP also produced a decrease of levels of myeloperoxidase. Pinocembrin (**4**) has been reported with neuroprotective, anti-oxidative, and anti-inflammatory effects both in vitro and in vivo [25,26]. Several biological activities [37,38], have been attributed to pinocembin (**4**), and pinostrobin (**1**), and support the pharmacological properties of 4-EAEP. 

## 4. Materials and Methods

### 4.1. Propolis

The propolis samples 1, 2, 3, and 5 were from local *Apis mellifera* bee breeders, while sample 4 proceeds from a wild colony, probably Africanized. All the samples were collected by one of the coauthors, Omar Argüello-Nájera, using the scraping technique, then ground in a coffee mill, and extracted with solvents with increasing polarity consecutively at room temperature. Each sample was extracted first with hexane, and then the residue with ethyl acetate and methanol. The exception was propolis 4, quite insoluble in hexane, but was completely dissolved in ethyl acetate, therefore this extract (4-EAEP) was the only one prepared. The solvents were eliminated in a rotatory evaporator, and the extracts stored in dark bottles at 4 °C. All the extracts were screened for their capacity to inhibit lipid peroxidation in the TBARS assay, the most potent was 4-EAEP, and therefore was selected to study its chemical composition, its anti-oxidant, anti-inflammatory and anti-mycobacterial activity. 

### 4.2. General Experimental Procedures

IR spectra were recorded on Tensor 27 spectrophotometer (Bruker, Billerica, MA, USA). NMR spectra were obtained on an Avance III 400 MHz or 700 MHz spectrometer (Bruker Billerica, MA, USA). EIMS were obtained on a MStation JMS-700 mass spectrometer (Jeol Ltd., Tokyo, Japan) and The AccuTOF JMS-T100LC (Jeol Ltd., Tokyo, Japan). CC (Sev-Prendo, Puebla, México) was carried out with silica gel 60 (Macherey-Nagel, Düren, Germany). SC was carried out with sephadex^TM^ LH-20 (GE Healthcare Bio-Sciences AB, Uppsala, Sweden). To monitor CC and SFpre-coated TLC-sheets ALUGRAM^®^Xtra SIL G/UV254 with silica gel 60, layer 0.20 mm (Macherey-Nagel, Düren, Germany).

### 4.3. Isolation Procedure of Compounds

The 4-EAEP (40 g) was fractionated by CC (hexane-ethyl acetate gradient), fractions with similar TLC patterns were combined. Compound **1** (118 mg) was obtained from fractions 39–40 eluted with a 95:5 mixture [39]. Compound **2** (18 mg) was obtained from fractions 89–99 eluted with a 90:10 mixture [40]. Compounds **3** (41 mg) and **4** (59 mg) were obtained from fractions 100–102 eluted with an 80:20 mixture [39]. Compound **8** (52 mg) was obtained from 237–240 eluted with a 60:40 mixture [41]. Compound **9** (10 mg) from fractions 265–272 eluted with a 60:40 mixture [42]. The pooled fractions 169–186 from the first column were next subjected to CC (hexane-ethyl acetate gradient). Compound **5** (11 mg) was obtained from subfractions 46–60 [43], **6** (16 mg) from subfractions 100–104 and **7** (25 mg) from subfractions 149–159 [44]. All compounds eluted with a 92:8 mixture. The pooled fractions 305–339 from the first column was next subjected to SC (eluted with 100% methanol). Compound **10** (8 mg) was obtained from subfractions 7–6. Compound **11** (9 mg) was obtained from subfraction 12 [45]. The identification of the isolated compounds (Figure 1) was achieved by comparison of their physical and spectroscopic data with those of the literature. 

Two new compounds, epoxypinocembrin chalcone (**6**), and a substituted ε-caprolactone **10** have not been previously reported, and their identification is thoroughly presented in the Results section.

*Pinostrobin* (**1**) White crystals, m.p. 87–90 °C. ^1^H-NMR (300 MHz, CDCl_3_). δ 12.02 (1H, s, 5-OH), 7.44 (5H, m, H-2′, H-3′, H-4′, H-5′ and H-6′), 6.08 (1H, d, *J* = 2.4, H-6), 6.07 (1H, d, *J* = 2.1, H-8), 5.42 (1H, dd, *J* = 3, 12.9, H-2), 3.81 (3H, s, OCH_3_-7), 3.09 (1H, dd, *J* = 12.9, 17.1, H-3a), 2.82 (1H, dd, *J* = 3, 17.1, H-3b). ^13^C-NMR (CDCl_3_): δ 195.89 (C-4), 168.14 (C-7), 164.31 (C-5), 162.94 (C-9), 138.53 (C-1′), 129.02 (C-3′, C-4′ and C-5′), 126.28 (C-2′ and C-6′), 103.31 (C-10), 95.30 (C-8), 94.42 (C-6), 79.38 (C-2), 55.83 (OCH_3_ C-7), 43.54 (C-3). ESI-MS (positive mode): *m*/*z* 271.09 [M + H]^+^. EI-MS: *m*/*z* (relative abundance) 270 [M^+^] (100), 269 [M − H]^+^ (50), 193 (55), 166 (42), 138 (17), 95 (14) [39,46]. 

*Izalpinin* (**2**) Yellow needles, m.p. 208–210 °C.^1^H-NMR (CDCl_3_). δ 11.65(1H, s, 5-OH), 8.2 (2H, m, H-2′, H-6′), 7.52 (3H, m, H-3′, H-4′, H-5′), 6.51 (1H, d, *J* = 2.2, H-8), 6.39 (1H, d, *J* = 2.2, H-6), 3.81 (3H, s, OCH_3_-7). ^13^C-NMR (CD_3_OD): δ 175.74 (C-4), 166.15 (C-7), 161.05(C-5), 157.21 (C-9), 145.38 (C-2), 136.75 (C-3), 130.89 (C-1′), 130.42 (C-4′), 128.77 (C-3′, C-5′), 127.77 (C-2′, C-6′), 104.19 (C-10), 98.19 (C-6), 92.43 (C-8), 56.03 (OCH_3_ C-7) [40]. ESI-MS (positive mode): *m*/*z* 285.07 [M + H]^+^. EI-MS: *m*/*z* (relative abundance) 285 [M + H]^+^ (18), 284 [M^+^] (100), 283 [M − H]^+^ (25), 241 (8), 105 (15), 77 (8) [18,40].

*Cinnamic acid* (**3**) White powder m.p. 133–135 °C ^1^H-NMR (CDCl_3_). δ 7.81 (1H, d, *J* = 15.9, H-3), 7.56 (2H, m, H-2′ and H-6′), 7.56 (3H, m, H-3′, H-4′ and H-5′), 6.46 (1H, d, *J* = 15.9, H-2). 177.62, 147.26, 134.2, 130.90, 129.11, 128.52, 117.46. EI-MS: *m*/*z* (relative abundance) 149 [M + H]^+^ (10), 148 [M^+^] (100), 147 [M − H]^+^ (95), 131 (28), 103 (42), 102 (25), 77 (33), 51 (17) [47].

*Pinocembrin* (**4**) Beige powder, m.p. 198–201 °C. ^1^H-NMR (300 MHz, CDCl_3_ + drop of DMSO-D6). δ 12.02 (1H, s, 5-OH), 7.42 (5H, m, H-2′, H-3′, H-4′, H-5′ and H-6′), 5.96 (1H, d, *J* = 2.1, H-6), 5.93 (1H, d, *J* = 2.1, H-8), 5.44 (1H, dd, *J* = 3, 12.6, H-2), 3.06 (1H, dd, *J* = 12.9, 17.1, H-3a), 2.77 (1H, dd, *J* = 3.3, 17.1, H-3b). ^13^C-NMR (CDCl_3_): δ 194.68 (C-4), 166.53 (C-7), 163.51 (C-5), 162.32 (C-9), 138.13 (C-1′), 128.17 (C-3′, and C-5′), 128.12 (C-4′), 125.72 (C-2′ and C-6′), 101.67 (C-10), 96.01 (C-6), 94.94 (C-8), 78.25 (C-2), 42.53 (C-3). ESI-MS (positive mode): *m*/*z* 257.08 [M + H]^+^. EI-MS: *m*/*z* (relative abundance) 256 [M^+^] (100), 255 [M − H]^+^ (63), 179 (80), 152 (65), 124 (34), 69 (15) [39,48].

*Kaempherol* (**5**) Yellow powder. ^1^H-NMR (CD_3_OD) δ 8.07 (2H, dd, *J* = 2.05, 8.85, H-2′ y H-6′), 6.9 (2H, dd, *J* = 2.05, 8.9, H-3′ and H-5′), 6.38 (1H, d, *J* = 2.05, H-8), 6.17 (1H, d, *J* = 2.1, H-6). ^13^C-NMR (CD_3_OD): δ 177 (C-4), 166.11 (C-7), 162.47 (C-5), 160.56 (C-4′), 158.31 (C-9), 148 (C-2), 137.09 (C-3), 130.66 (C-2′ and C-6′), 123.75 (C-1′), 116.32 (C-3′, C-5′), 104.41 (C-10), 99.47 (C-6), 94.6 (C-8) [43].

*Epoxypinocembrin chalcone* (**6**). White crystals, m.p. 99–102 °C. ^1^H- and ^13^C-NMR Table 1. ESI-MS (positive mode): *m*/*z* 273.07 [M + H]^+^. EI-MS: *m*/*z* (relative abundance) 273 [M + H]^+^ (8), 272 [M^+^] (40), 153 (100), 120 (20), 91 (28), 28 (16), 18 (16).

*3,3-Dimethylallyl caffeate* (**7a**). Crystalline light beige powder. ^1^H-NMR (CDCl_3_). δ 7.52 (1H, d, *J* = 16, Hα), 7.03 (1H, d, *J* = 1.6, H-2), 6.93 (1H, dd, *J* = 1.6, 8, H-6), 6.77 (1H, d, *J* = 8, H-5), 6.23 (1H, d, *J* = 16, Hβ), 5.4 (1H, tdq, *J* = 1.6, 2.8, 7.2, H-2′), 4.67 (2H, d, *J* = 7.2, H-1′), 1.77 (3H, s, H-5′), 1.75 (3H, s, H-4′). Compound **7a** was obtained as a mixture with *isopent-3-enyl caffeate* (**7b**). ^1^H-NMR (CDCl_3_). δ 7.52 (1H, d, *J* = 16, Hα), 7.03 (1H, d, *J* = 1.6, H-2), 6.93 (1H, dd, *J* = 1.6, 8, H-6), 6.77 (1H, d, *J* = 8, H-5), 6.23 (1H, d, *J* = 16, Hβ), 4.82 (1H, s, H-5), 4.77 (1H, s, H-5), 4.28 2H, t, *J* = 6.8, H-1′), 2.41 (2H, t, *J* = 6.8, H-2′), 1.78 (3H, s, H-4′) [44].

*3,4-Dimethoxycinnamic acid* (**8**). Crystalline white powder, m.p. 183–185 °C ^1^H-NMR (CDCl_3_) δ 7.73 (1H, d, *J* = 15.8, H-7), 7.14 (1H, dd, *J* = 2.1, 8.1, H-6), 7.07 (1H, d, *J* = 2, H-2), 6.88 (1H, d, *J* = 8.3, H-5), 6.32 (1H, d, *J* = 15.9, H-8), 3.92 (6H, s, OCH_3_). ^13^C-NMR (CD_3_OD): δ 172.5 (C-9), 151.2 (C-4), 149.4 (C-3), 147.1 (C-7), 127.2 (C-1), 123.2 (C-6), 115.0 (C-8), 111.2 (C-5), 110.22 (C-2), 109.99 (C-5), 56.1 (OCH_3_), 56.0 (OCH_3_). ESI-MS (positive mode): *m*/*z* 209.08 [M + H]^+^. EI-MS: *m*/*z* (relative abundance) 208 [M^+^] (100), 309 [M − H]^+^ (13), 193 (15), 147 (6), 133 (7), 119 (7), 91(9), 77 (10), 51 (7) [41].

*Rhamnetin* (**9**). Yellow powder. m.p. 282–287 °C. ^1^H-NMR (CD_3_OD): δ 7.76 (1H, d, *J* = 2.15, H-2′), 7.66 (1H, dd, *J* = 2.17, 8.47, H-6′), 6.89 (1H, d, *J* = 8.5, H-5′), 6.59 (1H, d, *J* = 2.2, H-8), 6.32 (1H, d, *J* = 2.2, H-6), 3.89 (3H, s, OCH_3_). ESI-MS (positive mode): *m*/*z* 317 [M + H]^+^. EI-MS: *m*/*z* (relative abundance) 316 [M^+^] (100), 317 [M − H]^+^ (21), 315 (13), 273 (12), 194 (16), 137 (12) [42].

*ε-Caprolactone derivative* (**10**). Beige amorphous powder, m.p. 87–90 °C. ^1^H and ^13^C-NMR Table 2. ESI-MS (positive mode): *m*/*z* 295.22 [M + H]^+^.

*Caffeic acid* (**11**). Slightly brown powder. m.p. ^1^H-NMR (CD_3_OD): δ 7.53 (1H, d, *J* = 15.87, H-7), 7.03 (1H, d, *J* = 2.1, H-2), 6.93 (1H, dd, *J* = 2.1, 8.1, H-6), 6.77 (1H, d, *J* = 8.1, H-5), 6.22 (1H, d, *J* = 15.87, H-8) ^13^C-NMR (CD_3_OD): δ 171.06 (C-9), 149.42 (C-4), 146.94 (C-7), 146.79 (C-3), 127.83 (C-1), 122.81 (C-6), 116.49 (C-5), 115.64 (C-8), 115.09 (C-2). ESI-MS (positive mode): *m*/*z* 181.05 [M + H]^+^. EI-MS: *m*/*z* (relative abundance) 181 [M + H]^+^ (12), 180 [M^+^] (100), 163 (39), 134 (41), 89 (21), 43 (18) [45].

### 4.4. HPLC Analysis of Propolis

The analysis was run on a Breeze HPLC-PDA system (Waters Corporation, Milford, MA, USA), equipped with degasser, 1525 binary pump and 2998 PDA. Separation was achieved on a Luna PFP(2) column (100 Å 250 mm × 4.6 mm, 5 µm particle size (Phenomenex, Torrance, CA, USA), at room temperature, using a gradient system composed by the binary phases (A) water and (B) acetonitrile, both with 0.1% of acetic acid. The elution gradient was: 0–3 min 25% B, 3–10 min 30% B, 10–40 min 40% B, 40–60 min 60% B and 60–92 min 90% B. The flow employed was 1 mL/min [49]. The injected volume was 20 μL. The concentration of the solutions depended of its λ maximum for each compound.

### 4.5. Animals 

The animals were provided by the Bioterio of IFC, UNAM, and their handling followed the Mexican Official Norm NOM-062-ZOO-1999 and international rules. The experimental procedures were approved by Ethic Committee (CICUAL-IQ-004-17). Groups of six CD1 male mice (25–30 g) were used for TPA assay and groups of three male Wistar rats (200–250 g) were used for TBARS. The animals were maintained under a 12 h light/dark cycle (22 °C ± 1 °C) with free access to food and water.

### 4.6. Inhibition of Lipid Peroxidation Measured by TBARS Assay

The tested compounds were **1**, **2**, **4**, **6**, **7a** + **7b** and **8**. Lipid peroxidation was measured by TBARS assays using rat brain homogenates. Three rats were sacrificed with CO_2_. Each whole brain was dissected and homogenized in PBS to produce a 1/10 (*w*/*v*) homogenate [50]. The homogenate was centrifuged for 10 min at 800 rcf, the supernatant protein content was adjusted with PBS at 2.66 mg of protein/mL. 375 µL of supernatant plus 50 µL of EDTA (20 µM) and 25 µL of each sample solved in DMSO were incubated at 37 °C for 30 min. Lipid peroxidation was started adding 50 µL of freshly prepared 100 µM FeSO_4_ solution, and incubated at 37 °C for 1 h. Concentration of TBARS was calculated by interpolation in a standard curve of TMP as a precursor of MDA [51]. Results were expressed as nmoles of TBARS per mg of protein. The inhibition ratio (I_R_(%)) was calculated using following formula I_R_ = (Abs_control_ − Abs_sample_) × 100/Abs_control_. BHT and α-tocopherol were used as positive standards.

### 4.7. Determination of Reactive Oxygen Species (ROS) in the Antigen-Induced Mast Cell Degranulation

The concentrations tested were selected based on preliminary assays with logarithmic scale. The tested compounds were **1**, **2**, **4**, **7a** + **7b** and **8**. The 4-EAEP was tested at 20, 200 and 2000 μg/mL; for **1** was 0.074–7.4 µM, for **2** was 0.035–3.5 µM; for **4** was 3–300 µM, for **7** was 1–100 µM and for **8** was 3–300 µM. Trolox was used as positive control (5 mM). The experiments were carried out with sensitized 2 × 10^6^ BMMC/mL [52] in a 1 mL by treatment, 15 min after add the compounds, the cells was stimulated, with the antigen DNP-HSA (27 ng/mL) and at the same time the indicator DCF-DA (10 μM) was added, 15 min after stop reaction at 4 °C. Then the tubes were centrifuged at 1500 rpm at 4 °C by 5 min, the supernatant was eliminated and 300 μL of Igepal (0.1%) was added at 37 °C, the mixture was pipetted to smash the cell bottom. Next, the tubes were centrifuged at 10,000 rpm by 5 min at 4 °C and 200 μL of supernatant were transferred at 96 well plate and red in Luminometer FLx88 (Biotek, Winooski, VT, USA), at 488 nm of λ_excitation_ and 565 nm of λ_emission_. The plate was red each 15 min during 1 h [34].

### 4.8. DPPH Scavenging Capacity

The activity of compounds **1**, **2**, **3**, **4**, **5**, **6**, **7a** + **7b** and **8** were assessed. Fifty μL of test compounds in ethanol at different concentrations was added to an ethanolic solution of DPPH (133.33 μM, 0.150 mL). Reaction mixtures were incubated at 37 °C for 30 min in the dark. After incubation the absorbance was measured at 515 nm in a microplate reader Synergy HT (BioTek Instruments, Winooski, VT, USA). The scavenging capacity (%) is calculated as [(Abs_control_ − Abs_sample_)/(Abs_control_) × 100]; α-tocopherol was used as standard [50].

### 4.9. Evaluation of Anti-Inflammatory Effect by TPA Assay

The effect of compounds **1**, **2**, **3**, **4**, **5**, **6**, **7**, **8** and **9** were tested, in the primary screening was determined that only the compound **4** had activity. Groups of five CD1 male mice were used, one group for vehicle, other group for reference drug (indometacin, Sigma, St. Louis, MO, USA) and four groups for each dose tested of pinocembrin (**4**). The animals were anesthetized with Pisabental (sodium pentobarbital, PISA, Ciudad de México, México). The right ear received topically TPA 2.5 µg in ethanol (10 µL), the left ear only ethanol. 10 min after in the right ear the test substances (1 μmol/ear), indomethacin (0.31 µmol/ear) or vehicle were applied. 4 h later, the mice were killed with CO_2_. From each ear was removed a 7 mm diameter plugs. The swelling was assessed as the difference in weight between right and left ear plugs [53]. The anti-inflammatory activity was expressed as inhibition of edema (IE): IE (%) = 100 − [B × 100/A], where A = edema induced by TPA alone, and B = edema induced by TPA plus sample. 

### 4.10. Myeloperoxidase (MPO) Assay

Tissue MPO activity was measured in biopsies taken from ears 4 h after TPA administration, only for vehicle, indometacin and compound **4** groups. Each biopsy was homogenized for 30 s at 4 °C. The homogenate was sonicated 20 s, and centrifuged at 12,000 rpm for 15 min at 4 °C. 10 μL of the supernatant were mixture with 180 μL of 80 mM PBS (pH 5.4) without HTAB at 37 °C. Then, 20 μL of 0.017% H_2_O_2_ and 20 μL of 18.4 mM 3,3′,5,5′-tetramethylbenzidine were added to start the reaction. Microtiter plates were incubated at 37 °C for 5 min. The reaction was stopped with the addition of 20 μL of H_2_SO_4_ (2M) and the Abs was measured a microplate reader using a BioTek Microplate Reader Synergy HT (BioTek Instruments, Winooski, VT, USA) at 405 nm. MPO activity was expressed as optical density/biopsy [54].

### 4.11. Histological Cuts

Histological cuts were obtained as described elsewhere [55]. Briefly, ear specimens were fixed in solution of 10% formalin. Ears were dehydrated, embedded in paraffin, and sectioned. Five-μm sections were stained with hematoxylin-eosin. Infiltration of cells was evaluated in selected areas (10× and 20× objective). 

### 4.12. Evaluation of Anti-Mycobacterial Activity by REMA (Resazurin Microtiter Assay)

*Mycobacterium tuberculosis* H37Rv ATCC 27294 were cultivated in 7H9-glycerol-10% ADC-0.01% tyloxapol médium at 37 °C until reach 0.4 OPD. Stock solution of 4-EAEP was prepared in DMSO (10 mg/mL) and rifampin were used as reference drug (16–0.001 mg/mL). Plates were incubated for 6 days, and then 30 µL of 0.01% resazurin sodium salt (Sigma-Aldrich, St. Louis, MO, USA) were added to each well and re incubated for 2 days more. Colors were interpreted visually as blue (no growth), pink (growth) and MIC as the last well in which blue color were observed [47].

### 4.13. Evaluation of Cytotoxicity in Vero Cells

Vero cell line from ATCC^®^ CCL-81^TM^ were cultured in RPMI 1640 medium (10% of FBS and non-essential aminoacids). 10,000 Vero cells per well were incubated for 24 h in 100 µL of RPMI medium. After that, these cells were washed and new fresh medium with compounds at different concentrations was added and incubated for 48 h (37 °C and 5% CO_2_ atmosphere), after, 10 mL of MTT (5 mg/mL) were add to each well and re incubated for 4 h. The medium was removed and 100 µL of DMSO were used to solubilize the formazan. Abs were determinate at 570 nm and cytotoxicity were calculated as % = (1 − (Abs_sample_/Abs_control_)) × 100 [56]. 

### 4.14. Statistical Analysis

All data were represented as mean ± standard error (SEM). Data were analyzed by one-way ANOVA followed by Tukey’s test. IC_50_, was estimated by means of a linear regression.

## 5. Conclusions

We reporting two new compounds, epoxypinocembrin chalcone, and an ε-caprolactone derivative, as well as nine known compounds isolated from a red propolis from Chiapas, México. The propolis extract showed high capacity to prevent lipid peroxidation, being the active principles 3,3-dimethylallyl caffeate and isopent-3-enyl caffeate. Moreover, the extract showed an anti-oxidant activity at low concentrations in two models, mainly associated with izalpinin and pinocembrin, since these reduced the high ROS levels of mast cells, induced by the cross-linking of the antigen with the IgE antibody and its high affinity receptor FcεRI, that are involved in acute allergic inflammatory reactions. Also, the propolis extract and pinocembrin showed anti-inflammatory activity in the model of TPA dependent on the activation of neutrophils and macrophages. For this reason, this study supports that the use of standardized propolis extracts could be useful for the treatment of chronic degenerative inflammatory processes such as allergy and asthma.

## Figures and Tables

**Table 1:** Propolis from México and screening activity of their extracts in TBARS assay.

Propolis Sample/Location (Altitude), State	Extract	%Inhibition (10 µg/mL)	IC_50_ (μg/mL)
**1** Tapachula (177 m), Chiapas	Hexane	1.32	
	Ethyl acetate	5.28	
	Methanol	9.23	
**2** La Trinitaria (1558 m), Chiapas	Hexane	52.58	46.17 ± 0.83
	Ethyl acetate	94.68	8.11 ± 0.16
	Methanol	96.11	11.17 ± 0.61
**3** Pantelhó (1056 m), Chiapas	Hexane	21.37	
	Ethyl acetate	35.28	25 ± 1.39
	Methanol	64.00	11.59 ± 88
**4** San Cristóbal de las Casas (2200 m), Chiapas	Ethyl acetate (**4-EAEP**)	99.28	1.42 ± 0.07
**5** Rancho San Pedro Chenchelá (227 m), Yucatán	Hexane	12.06	
	Ethyl acetate	23.33	
	Methanol	20.47	

**Table 2:** ^1^H- and ^13^C-NMR chemical shifts of epoxypinocembrin chalcone (**6**).

Position	δ_H_ (*J* in Hz)	δ_C_	Position	δ_H_ (*J* in Hz)	δ_C_
**1**		138.55	**1′**		101.81
**2**	7.53 dd (7,2.1)	128.92	**2′**		165.34 ^a^
**3**	7.41 m	129.4	**3′**	5.94 d (2.1) ^b^	97.45 ^b^
**4**	7.39 m	129.9	**4′**		168.84
**5**	7.41 m	129.4	**5′**	5.90 d (2.1) ^b^	96.35 ^b^
**6**	7.53 dd (7,2.1)	128.92	**6′**		164.38 ^a^
**7**	5.07 d (11.5)	85.04			
**8**	4.54 d (11.5)	73.71			
**9**		198.18			

^a^, ^b^ Assignments may be reversed.

**Table 3:** ^1^H- and ^13^C- NMR chemical shifts of ε-caprolactone derivative.

Position	δ_H_ (*J* in Hz)	δ_C_	Position	δ_H_ (*J* in Hz)	δ_C_
**1**		179.9	**10**	6.00 dd (11.1, 11.1)	129.43
**2**	2.21 t (7.6)	36.70	**11**	5.41 dt (11, 7.7)	133.08
**3**	1.59 q (7.1)	26.75	**12**	2.19 td (8.05,7.6)	28.63
**4**	1.53 m	33.75	**13**	1.39 q (7.14)	30.55
**5**	1.34 m	30.5	**14**	1.28–1.36 m	30.48
**6**	3.48 m	75.69	**15**	1.28–1.36 m	26.83
**7**	3.96 dd (7,6)	76.9	**16**	1.28–1.36 m	32.51
**8**	5.73 dd (15, 7)	133.53	**17**	1.28–1.36 m	23.61
**9**	6.56 ddt (15, 11.1, 1.1)	128.37	**18**	0.9 t (7)	14.4
